# A Hybrid System of Braden Scale and Machine Learning to Predict Hospital-Acquired Pressure Injuries (Bedsores): A Retrospective Observational Cohort Study

**DOI:** 10.3390/diagnostics13010031

**Published:** 2022-12-22

**Authors:** Odai Y. Dweekat, Sarah S. Lam, Lindsay McGrath

**Affiliations:** 1 Department of Systems Science and Industrial Engineering, Binghamton University, Binghamton, NY 13902, USA; 2 Wound Ostomy Continence Nursing, ChristianaCare Health System, Newark, DE 19718, USA

**Keywords:** pressure ulcer, pressure injures, hospital-acquired pressure injuries, HAPI, integrated system, Braden Scale, machine learning, genetic algorithm, support vector machine, diagnosis

## Abstract

**Background**: The Braden Scale is commonly used to determine Hospital-Acquired Pressure Injuries (HAPI). However, the volume of patients who are identified as being at risk stretches already limited resources, and caregivers are limited by the number of factors that can reasonably assess during patient care. In the last decade, machine learning techniques have been used to predict HAPI by utilizing related risk factors. Nevertheless, none of these studies consider the change in patient status from admission until discharge. **Objectives:** To develop an integrated system of Braden and machine learning to predict HAPI and assist with resource allocation for early interventions. The proposed approach captures the change in patients’ risk by assessing factors three times across hospitalization. **Design:** Retrospective observational cohort study. **Setting(s):** This research was conducted at ChristianaCare hospital in Delaware, United States. **Participants:** Patients discharged between May 2020 and February 2022. Patients with HAPI were identified from Nursing documents (N = 15,889). **Methods:** Support Vector Machine (SVM) was adopted to predict patients’ risk for developing HAPI using multiple risk factors in addition to Braden. Multiple performance metrics were used to compare the results of the integrated system versus Braden alone. **Results:** The HAPI rate is 3%. The integrated system achieved better sensitivity (74.29 ± 1.23) and detection prevalence (24.27 ± 0.16) than the Braden scale alone (sensitivity (66.90 ± 4.66) and detection prevalence (41.96 ± 1.35)). The most important risk factors to predict HAPI were Braden sub-factors, overall Braden, visiting ICU during hospitalization, and Glasgow coma score. **Conclusions:** The integrated system which combines SVM with Braden offers better performance than Braden and reduces the number of patients identified as at-risk. Furthermore, it allows for better allocation of resources to high-risk patients. It will result in cost savings and better utilization of resources. **Relevance to clinical practice:** The developed model provides an automated system to predict HAPI patients in real time and allows for ongoing intervention for patients identified as at-risk. Moreover, the integrated system is used to determine the number of nurses needed for early interventions. **Reporting Method:** EQUATOR guidelines (TRIPOD) were adopted in this research to develop the prediction model. **Patient or Public Contribution:** This research was based on a secondary analysis of patients’ Electronic Health Records. The dataset was de-identified and patient identifiers were removed before processing and modeling.

## 1. Introduction

Pressure-related skin damage results in injuries that are painful, disfiguring, and costly to treat for individuals who experience these types of injuries. A pressure injury is defined by the National Pressure Injury Advisory Panel (NPIAP) as “localized damage to the skin and underlying soft tissue usually over a bony prominence or related to a medical or other device” [1]. Pressure injuries are further broken down into two categories: Hospital-Acquired Pressure Injuries (HAPI) or Community-Acquired Pressure Injuries (CAPI). HAPI (also known as bedsores or pressure ulcers) are considered rare events by the Centers for Medicare and Medicaid services; hospital HAPI rate is reported, and hospitals can face financial penalties as well as reduction in hospital grades [1]. HAPI prevention has become a national focus but identifying at-risk patients and individualizing care can be costly, labor intensive, and potentially burdensome for the patient who receives care as well as those who provide the care.

**Figure 1:**
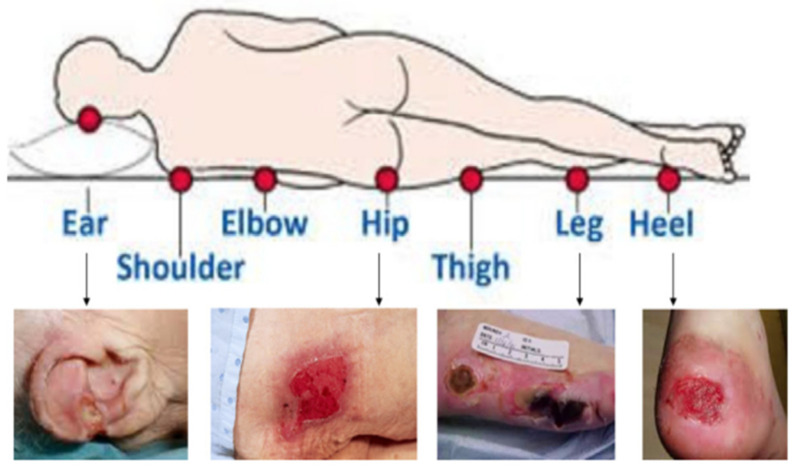
Different locations of HAPI [4].

Prevention of pressure injuries is an important foundation in safe patient care. Prevention must consider a holistic view of the patient because pressure injuries can occur anywhere on the body that is subjected to pressure or pressure in combination with shear, while factoring in the patient’s overall tissue tolerance [2,3], as shown in Figure 1. If HAPI occurs, the injury will be staged based on the type of tissue noted in the wound bed; these injuries will increase Length-of-Stay (LOS), require increased resources, financial penalties, and if the injury extends beyond the dermis, repeat injuries to the area will be a lifelong struggle for the patient. Early identification of at-risk patients as well as an individualized care plan for pressure injury prevention can reduce the HAPI rate.

The majority of HAPI can be prevented through risk assessment and targeted prevention interventions; however, most patients in acute care or long-term care settings are at risk, and the cost of prevention in terms of labor and preventive products can be significant. The best way to manage HAPI is to prevent them from happening. The first step when an individual enters the inpatient setting is to use a pressure injury risk assessment tool [1].

Identifying individual risk of early development of HAPI allows the caregiver to implement prevention techniques to reduce the risk of increased mechanical load and increase tissue tolerance. The earlier the risk is identified the less likely the patient will develop HAPI. Using a standardized risk assessment can help narrow down the volume of patients at risk for pressure injury development and allows to identify modifiable risk factors specific to the patient. Furthermore, it allows the care provider to develop a care plan and put specific risk control interventions in place, but it still leaves a large quantity of patients at risk.

Over 100 unique risk factors have been identified as potentially contributing to development of a pressure injury. Factors can be broken down into two categories: factors that affect tissue tolerance or factors that lead to increased mechanical load. These risk factors can be further broken down into modifiable or nonmodifiable. The sheer number of risk factors place most hospitalized patients at risk for pressure injury development; a focus on preventing these injuries can be overwhelming in the setting of reduced resources and low staffing volumes [1].

Several pressure injury risk screening and assessment tools are available, which classify patients into risk categories; these include the Braden Scale, Norton Scale, Waterlow Score, Cubbin-Jackson Scale, Braden Q Scale, and Spinal Cord Injury Pressure Ulcer Scale (SCIPUS) [5,6,7,8,9,10,11,12].

Table 1 summarizes the specialties, advantages, disadvantages, and risk factors for the most common risk assessment tools: Braden Scale, Norton Scale, Waterlow Score, Cubbin-Jackson Scale, Braden Q Scale, and SCIPUS. Table 1 visually addresses gaps associated with each method. For example, the Norton Scale can be used for general purposes but focuses mainly on orthopedics. It has only five primary subscales: activity status, mobility status, nutrition status, continence, and physical and mental conditions. However, Norton does not consider any other factors that Braden covers, such as friction, shear skin moisture, and sensory perception. On the other hand, physical and mental conditions are covered under the Norton scale but were not addressed by Braden. Risk assessment covers many modifiable risk factors. However, none of the risk assessment tools address all of the modifiable risk factors, nor do they consider non-modifiable risk factors. Critically ill patients and other special populations have additional risk factors that are not considered in the Braden Scale.

The one most often used is the Braden Scale [8,10,11,12,13,14,15,16,17,18,19]. Braden scale has six different subscales. Each subscale ranges from 1 (highest risk) to 4 (lowest risk) except for friction and shear (1–3). Therefore, the possible score range is from 6 to 23. The total score for a patient is the summation of scores for each subscale. In this scale, if the total score is less than or equal to 18, then the patient is predicted as an at-risk patient to develop HAPI; otherwise not considered to be at risk [13].

Risk assessment tools are used as a guide to help caregivers identify risk; however, they are limited by the number of factors that a caregiver can reasonably assess during patient care. These assessments also identify a large percentage of hospitalized patients as being at risk for developing pressure injuries. The disadvantage of using standardized risk assessments is the large percentage of at-risk patients who require extra interventions aimed at a large percentage of the population, who may not actually develop a pressure injury. The drawbacks and limitations of the risk assessment tools are as follows: There is no general model that considers all the above risk factors in one model. Furthermore, it has relatively high False Positive Rate (FPR) (suspect the wrong target). Providing interventions to wrong target where high type 1 error, which means high intervention costs. Moreover, risk assessments do not consider several risk factors such as weight loss, body temperature, Glasgow coma score, artificial ventilation, parenteral nutrition, American Society of Anesthesiologists (ASA) score, and many other factors.

Adding in additional components of machine learning can narrow down the volume of at-risk patients. There is a significant need to utilize machine learning to identify the total level of risk and using that information to target resources to those most at risk.

HAPI has received a lot of attention from researchers in the past few years through artificial intelligence because this is a quality-of-care measure that impacts patient care outcomes and healthcare professionals’ responsibilities [20].

This paper is organized as follows: Section 2 summarizes the contribution of this research. Section 3 summarizes and criticizes the related literature in predicting HAPI. Section 4 describes the study design and setting, participants, data source, variables, model development, statistical analysis, and ethical considerations. Section 5 summarizes the results of the developed method and compares it with Braden Scale. Section 6 discussed the output of the suggested results, their implications for the new approach in the medical field, the economic impact of the suggested model and limitations of this research. Lastly, Section 7 provides the conclusion and future work of this research.

## 2. Contributions

**What is already known?**

- The Braden Scale, a structured risk assessment for predicting pressure injury risk, identifies a large percentage of hospitalized patients as at-risk, but it does not consider nonmodifiable risk factors in predicting risk.
- Machine learning is used to predict hospital-acquired pressure injuries by incorporating a large group of modifiable and non-modifiable risk factors as well as considering changes over time; currently not considered by other studies.
- No retrospective observational cohort studies merged Braden Scale with a machine learning model to predict HAPI by incorporating multiple risk factors.

**What does this paper contribute to the wider global clinical community?**

- This research integrates machine learning with Braden Scale in one model to predict HAPI and identify additional risk factors that contribute to the development of pressure injuries.
- This study considers the change in patient conditions/vitals from admission until discharge by assessing relevant factors three times during hospitalization (first, last, and average values) rather than using a single data snapshot.
- The proposed model is used to determine the number of nurses needed for early interventions by utilizing the Electronic Health Record (EHR) and without increased workload on the care team.

## 3. Related Literature

This literature review focused on studies that proposed predictive models to determine patients’ HAPI risk to drive early interventions to prevent high-risk individuals from developing HAPI. After an extensive review of the literature from 2007 until the present, 24 studies were found that proposed HAPI risk prediction model using machine learning algorithms. The studies included in this literature review are retrospective observational studies published within the last 10 years and it developed a predictive model using machine learning to predict HAPI, which means that the data was collected for a historical timeframe and analyzed using machine learning algorithms to explore insights about patients who had already developed HAPI during their stay. The authors of this research focused on publications that utilized a highly imbalanced dataset where HAPI rate is less than or equal to 5% and that falls under the definition of a rare event, because HAPI are an adverse event that happen among a minority sample of patients. Therefore, there are six studies that utilized machine learning in predicting HAPI [21,22,23,24,25,26].

One of the most important factors to be considered when building HAPI risk prediction model is to compare the model’s performance to what is currently being used by wound care teams: nurse assessments for pressure injuries. Having a benchmark with which to compare proposed models provides additional strength to the model and confidence from model users and adopters. Only one out of six studies compared the results of proposed predictive models to the Norton Scale [24]. However, Norton risk factors were not included as model inputs in their study. There was an increase in the machine learning performance metrics compared to the Norton Scale; sensitivity increased by 5.88%, specificity increased by 15.62%, and the Area Under the receiver operating characteristic Curve (AUC) increased by 18.66% [24].

Using balancing techniques could impact the performance of trained predictive models. There is a significant variation in the machine learning algorithms used to build HAPI predictive models in the literature combined with data balancing techniques such as random sampling [22] and Synthetic Minority Oversampling Technique (SMOTE) [24]. The main disadvantage of the oversampling technique is that it duplicates the minor existing rare sample (cases with HAPI), which could increase the training performance in the model (i.e., overfitting). Consequently, the sensitivity of capturing the HAPI cases could be low in the implementation phase after developing the model. For example, the AUC of Nakagami et al. (2021) model was 80.0%, whereas the HAPI rate was 0.5%. Similarly, the AUC of the Ladies-Martin et al. (2020) model was 88.00%, which had a 4% HAPI rate.

In all six studies, the AUC was used to evaluate the models’ performance in addition to sensitivity and specificity. None of the six studies reviewed used integrated systems of machine learning and clinical risk assessment method as one of the model inputs to deliver higher accuracy and performance. In other words, no studies based on this inclusion criterion used risk assessment factors with other risk factors as input variables to the machine learning model to predict HAPI.

Furthermore, all the proposed models in the literature were static models that did not consider the changes in patient data from admission until discharge, because those models were built and tested on static datasets that did not change across patient stays; static data represent a summary snapshot of the data for the patient stay regardless of how many days they spent in the hospital.

It can be concluded from the reviewed literature that there is a need for a model that can utilize dynamic data to reflect the changes that happen to the patient during the hospital stay. Reliance on a snapshot of the data does not represent reality, because patients who develop HAPI have changing conditions and vitals across their stay that highly impact their risk of developing HAPI. Furthermore, there is a need for an integrated system that merges nursing scales as an input variable to the machine learning model to improve the prediction accuracy of HAPI.

This research aims to fill the literature gap by combining Braden Scale and machine learning model into one integrated model to predict HAPI and collect variables at multiple points. Moreover, the integrated model can be used to determine the number of nurses needed for early interventions by utilizing the EHR. This research describes the development and validation of the proposed model. EQUATOR guidelines for transparent reporting of a multivariable prediction model for individual prognosis or diagnosis (TRIPOD) were applied [27].

## 4. Methods

### 4.1. Study Design and Setting

This research is a retrospective observational cohort study conducted in ChristianaCare hospital. ChristianaCare, headquartered in Wilmington, Delaware, US, includes three hospitals (1430 beds) with an extensive network of outpatient and inpatient services. The hospital is a not-for-profit teaching health system with more than 260 residents and fellows.

### 4.2. Participants

This study includes admitted and discharged patients between May 2020 and February 2022 (42,861 patients). Pediatric patients less than 18 years of age, labor and delivery, and Emergency Department (ED) patients were excluded from this study. In addition, patients with less than three days of LOS were excluded from this study. It was noticed that none of the patients with LOS less than or equal to three days had HAPI, which therefore excludes these from the dataset reduces the number of samples for non-HAPI patients and increases chances to predict HAPI with high accuracy. As a result of this, the number of participants in this study was 15,889 patients, whereas only 485 patients had HAPI.

### 4.3. Data Source

Electronic health record was the main source for data extraction; variables were pulled from multiple SQL tables. Patients with HAPI were identified through Wound, Ostomy, Continence (WOC) nurse documentation, which included patient information with injury details. Variables were connected from multiple sources that included WOC validation files to provide a complete set of variables across different points of time during the patient visit. This resulted in a dataset that has a unique record per patient visit with variables obtained from historical visits if there were any. Pressure injuries that existed on admission or were community acquired were not included in the definition of HAPI because this work focused on injuries acquired only in the hospital.

### 4.4. Variables

The outcome of this study is to predict whether a patient will develop HAPI or not based on the symptoms and risk factors for each patient. The Braden scale focuses on six modifiable risk factors, which include factors that affect tissue tolerance as well as factors that lead to increased mechanical load [1]. The Braden scale can be used across the health care spectrum and has specific criteria to assist the nurse or health care provider to assign a score in each of the six categories: activity status, friction and shear status, mobility status, nutrition status, sensory perception status, and skin moisture status [1,10].

Other demographical, medical, diagnosis, labs, medications, medical devices, and risk assessment factors not considered in Braden are identified and scored using the EHR. In this study, 98 risk factors are used as input for the machine learning model, which include Braden Scale risk factors. The risk factors that potentially contribute to the development of HAPI were captured based on the literature review of other studies [1,21,22,23,24,25,28,29,30,31,32,33,34,35,36,37]. The medical team and WOC Nurses added or adjusted some other risk factors. Then, the EHR experts evaluate the availability of the suggested risk factors, which end with the 98 risk factors (Table S1 in supplementary material).

To capture the changing status of patients during their stay, dynamic risk factors are assessed at three times: risk factors/diagnosis upon admission (First), most recent diagnosis before discharge (Last), and the average of all observed values during visit (Average). Other risk factors that denoted the historical records or other static risk factors, such as race, however, are only considered once in this model.

### 4.5. Model Development

Braden scale is calculated for each patient; as discussed, if the total score is less than or equal to 18, then the patient is predicted to be an at risk HAPI patient, otherwise not at risk. Results of the first Braden Scale are measured at admission, whereas the most recent Braden Scale results are measured before discharge.

Machine learning is a unique process, where a large pool of patient information is fed into an algorithm to look for similarities and patterns in conditions. In the case of HAPI historical patient data is fed into the algorithm and variables are identified, which affects both HAPI and risk factors (i.e., training process). When given adequate data, the variables can be applied to current and future patients to identify which patients have similar variables to historical patients who went on to develop pressure injuries (testing process). The risk score (i.e., probability of a specific patient developing HAPI) can be turned into a level of risk and alerts to the care team. Machine learning is precise to the population studied; the variables identified are specific to acute care patients who experience HAPI. Whereas the specific variable may not cross the healthcare continuum into the outpatient or ambulatory setting, the machine learning prediction model is given all above 98 risk factors of the population to be studied and learn the hidden relationship for developing HAPI.

Support Vector Machine (SVM) is adopted to construct this study’s machine learning prediction model. SVM is a supervised machine learning algorithm that is commonly used in HAPI prediction and pattern recognition [22,23,24,26,32,35,38,39]. It has a high generalizing capacity. Because the data is highly unbalanced (only 3% of patients had HAPI), there is a need for a technique to deal with this issue.

Genetic Algorithm (GA) is an evolutionary algorithm used to optimize the hyperparameters of SVM in this study [40]. Cost-sensitive learning is a well-known approach in machine learning that deals with unbalanced class distribution by assigning different weights/costs to the types of misclassification errors during the training process [41,42]. The hyperparameters of SVM are the cost-sensitive learning factors associated with each misclassification error to minimize the total misclassification cost.

Data preprocessing is a crucial phase before developing machine learning prediction models. It modifies the data to a suitable format that is readable by the machine learning algorithm. Single imputation, normalization, and categorization for categorical factors using dummy variables are used in this regard. The single imputation method ensures that the missing data for some missing records of patients is filled.

Recursive Feature Elimination (RFE) is used as a feature section tool, which fits a prediction model and removes the weakest risk factors until the optimal number of risk factors is reached [43]. Risk factors that are selected by RFE are in gray color in the supplementary Table S1 (63 risk factors out of 98). First, last, and average of the overall Braden Scale and all six different subscales are considered by RFE as dominant factors in this model. To evaluate the influence of each risk factor to predict HAPI, feature importance was determined using Gini impurity in random forest.

**Figure 2:**
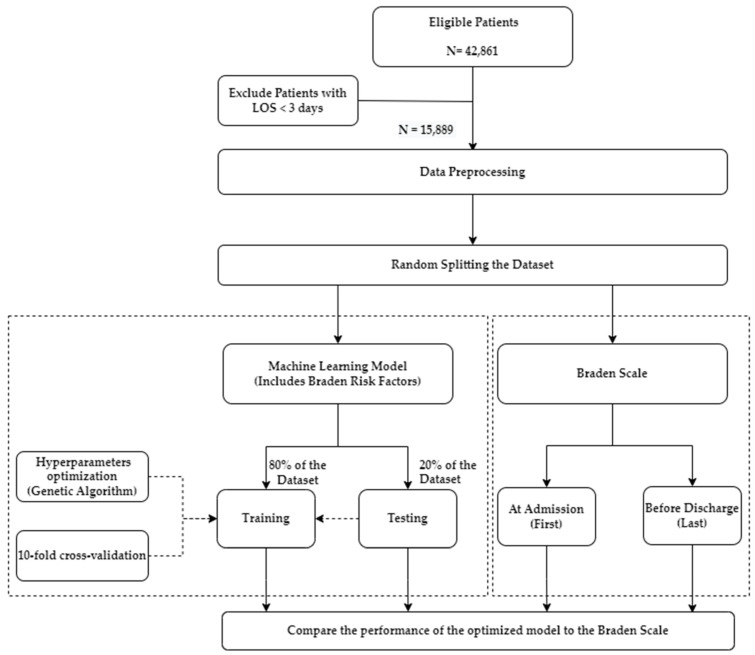
Research framework.

The dataset of 15,889 patients is divided into 80% for training the model with 10-fold cross-validation and the proposed hyperparameter method. The remaining 20% is used to test the model’s performance on unseen patients with HAPI and without HAPI. The optimized hyperparameters are used again for 50 experiments; in each experiment, the distribution of splitting the 80% vs. 20% is changed randomly to ensure the algorithm is robust on different 50 distributions. The exact distribution of 50 experiments is used when constructing the Braden Scale to ensure a fair comparison (i.e., using same seeds when splitting the dataset). The separation compares the same patients’ results under the same circumstances in both models (optimized SVM vs. Braden Scale). The research framework of this study is summarized in Figure 2.

RStudio 2022.02.2 is used for data preprocessing and SQL server management studio is used for data acquisition. The machine learning models and experimentation with Braden Scale were developed using Python 3.9.0.

The performance of the training sets vs. the testing sets is compared to ensure no overfitting in the model. Performance metrics are sensitivity, specificity, AUC, FPR, and detection prevalence. The confusion matrix output determines these metrics: False Negative (FN), False Positive (FP), True Negative (TN), and True Positive (TP). FN is the number of incorrect predictions that a patient does not have HAPI, i.e., HAPI is identified as non-HAPI. FP is the number of incorrect predictions that a patient has HAPI, i.e., non-HAPI is detected as HAPI. TN is the number of correct predictions that a patient does not have HAPI, i.e., non-HAPI is correctly classified as non-HAPI. TP is the number of correct predictions that a patient has HAPI, i.e., HAPI is correctly classified as HAPI [44,45].

Sensitivity refers to the ratio that the model correctly classifies TP HAPI cases out of all cases with HAPI. Specificity measures how well it can predict patients as not having HAPI out of those diagnosed without HAPI. Detection prevalence indicates the percentage of patients predicted as high risk by the model. This indicates the resources needed to provide prevention actions for the suspected HAPI cases (i.e., TP and FN) by WOC nurses. FPR measures the probability of non-HAPI cases predicted as HAPI cases. Lastly, AUC measures the ability of the model to distinguish between patients with and without HAPI.

### 4.6. Statistical Analysis

The statistical analysis, conducted in Minitab 20, compares risk factors that affect patients with HAPI vs. patients without HAPI. T-test is performed for continuous risk factors (when it has N/A for sub-factors in the supplementary Table S1), and the chi-square test is conducted for categorical risk factors (when it has sub-factors in the supplementary Table S1). The risk factors that are statistically significant when comparing HAPI patients to non-HAPI patients have *p*-value < 0.05 represented in bold in the same table.

### 4.7. Ethical Considerations

This research was based on a secondary analysis of EHRs at ChristianaCare hospital in Delaware, United States. The ChristianaCare hospital Institutional Review Board (IRP) approved the study without written informed consent from participants. The dataset was de-identified where patient identifiers were removed before processing and modeling.

## 5. Results

In this study, the HAPI rate is 3% (485 out of 15,889 patients), which is highly unbalanced for developing a classical machine learning prediction model for HAPI before occurrence. A comprehensive 98 risk factors, which include Braden assessment factors, are collected for each patient using EHR to construct a machine learning prediction model; some of the dynamic risk factors are taken at three points of time (First: at admission, Last: the Most Recent Braden Scale before discharge, and Average: the average between the first and most recent). GA is used as an optimization tool to optimize the hyperparameters of SVM using 10-fold cross-validation. The dataset is divided into 80% for training the model with 10-fold cross-validation and the remaining 20% is used to test the model’s performance. The optimal hyperparameters of the proposed model are used for 50 experiments; in each experiment, the dataset is randomly distributed into 80% for model development and 20% for testing.

In addition, the Braden Scale is measured and compared for the same set of patient records. Two models of Braden Scale are developed: First Braden Scale at admission and the Most Recent Braden Scale before discharge. Then, the proposed model is compared to the Braden Scale in terms of sensitivity, specificity, FPR, AUC, and detection prevalence. The same random separations between training and testing were adopted for Braden models to enable a fair comparison under the same patients in every experiment. The 95% confidence interval is calculated as shown in Table 2.

The optimized SVM achieved the highest sensitivity (74.29 ± 1.23), specificity (77.29 ± 0.37), AUC (75.79 ± 0.58), FPR (22.71 ± 0.37), and detection prevalence (24.29 ± 0.35) for the testing set, when compared to the two Braden Scale models. Similar results are observed for the optimized SVM in the training set.

The supplementary Table S1 summarizes the characteristics of the risk factors for two groups of patients in this study: HAPI vs. non-HAPI patients. The risk factors that have *p*-value in bold are statistically significant when comparing HAPI patients to non-HAPI patients. Results indicate that all Braden’s risk subgroups and other factors are statistically significant (*p*-value < 0.05). On the contrary, risk factors that are colored in gray in the supplementary Table S1 are those selected by the machine learning system.

**Figure 3:**
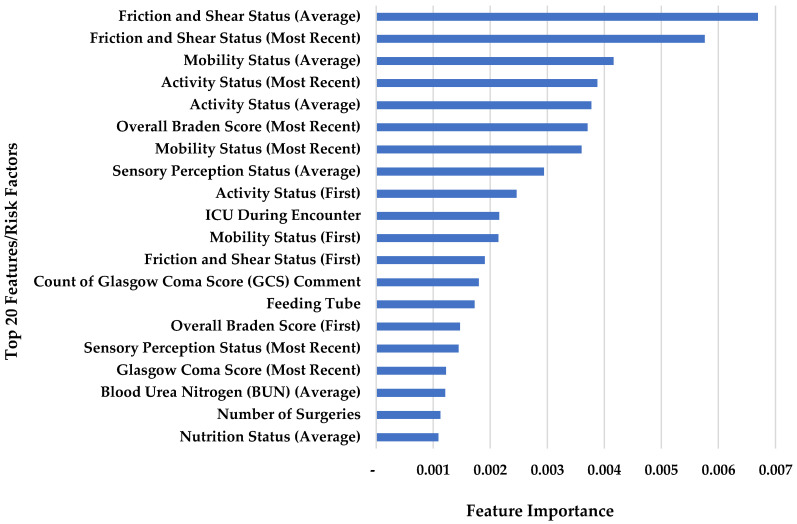
Feature importance for the top 20 risk factors that affect HAPI.

To evaluate the influence of each risk factor on predicting HAPI, feature importance was determined. Figure 3 illustrates the importance of the top 20 risk factors in predicting HAPI. The most important factors are Braden sub-factors and overall Braden, visiting ICU during hospitalization, count of Glasgow Coma Score (GCS) comment, feeding tube, GCS, Blood Urea Nitrogen (BUN), and number of surgeries.

## 6. Discussion

This research introduces an integrated and robust Braden Scale and machine learning system to predict HAPI. The outcome of this research will help determine the number of nurses needed for early interventions. This study used the most dominant risk factors, which include the Braden scale, and factors not considered in Braden, such as demographical, medical, diagnosis, labs, medications, and medical devices. The proposed approach captures changing patient data/diagnoses by assessing factors three times during hospitalization (first, last, and average) rather than using a static data snapshot.

The overall Braden score and five out of six Braden’s subgroups (activity status, friction and shear status, mobility status, nutrition status, and sensory perception status) are among the most significant risk factors in the proposed model. Similarly, those factors are statistically significant when comparing HAPI patients vs. non-HAPI patients. This implies that the Braden risk score is a robust tool for predicting HAPI. Furthermore, the proposed integrated system does not remove components from Braden but adds other factors that are not considered in Braden scale into one solid model.

Unlike other studies, which relied on a snapshot of the data (either at admission or at a single point of time), the proposed approach incorporates the changes in patient data/diagnoses, as patients’ conditions and vitals can vary during their hospital stay. Inconstant/ unsteady risk factors are assessed three times: upon admission (First), most recent before discharge (Last). Third, the average of all observed values during visit (Average).

The integrated model achieves a fairly good AUC (75.79 ± 0.58) compared to the First Braden Score alone (62.31 ± 3.13). Some studies applied a machine learning model to predict HAPI without considering Braden reached AUC more than this study. For example, the AUC was 89.70, 89.00, 80.40, 94.00, and 90.00 for [21,22,23,24,25,26], respectively. However, those studies adopted oversampling techniques to improve the AUC. This study uses cost-sensitive learning which penalizes misclassification in the minor class to establish a sustainable model. Unlike other studies, length of stay was not considered a risk factor in this study because data analysis showed that HAPI drive a longer LOS. Lastly, all locations of injuries were included in the scope of this study.

Clinically, the use of an integrated model of Braden scale and machine learning allows greater allocation of resources to the patients. Using the First Braden Scale alone predicted 41.96 ± 1.35% patients to be at risk for pressure injury development during their hospital stay (i.e., detection prevalence) with a sensitivity of 66.90 ± 6.58. With this large percentage of the population defined as at-risk, resource allocation for the prevention of pressure injuries is spread thin and individualizing care plans to meet the patient needs becomes challenging. With the addition of the Braden scale through SVM only 24.29 ± 0.35% patients were identified as at risk for pressure injury development with a sensitivity of 74.29 ± 1.23. Nevertheless, based on acuity and care needs associated with the Braden, the resources available to the care team may not be adequate to meet the needs of all patients.

The reduction in the number of predicted at-risk patients allows for greater allocation of available resources to prevent the maximum number of patients from developing HAPI. The smaller number of patients predicted would allow local nursing leadership or charge nurse to modify plan of care and provide additional support from the direct care team.

From WOC nurses’ perspective, a focus on prevention of injuries is a key component of the WOC nurse knowledge base and training; however, the clinical focus shifts to treatment of these areas given the sheer volume of hospitalized patients with wounds.

**Figure 4:**
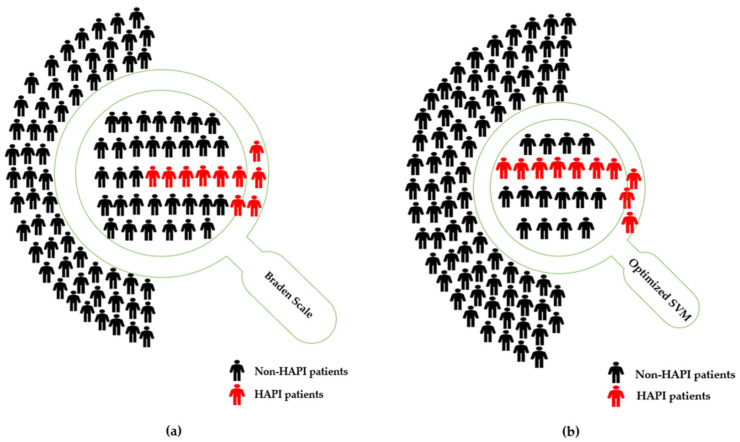
(**a**) Illustrate a sample of 100 patients, where 10 of them have HAPI. **Braden Scale alone**: conduct prevention actions for 42 patients (i.e., detection prevalence), predict 6.6 patients with HAPI (i.e., sensitivity) correctly; (**b**) Illustrate a sample of 100 patients, where 10 of them have HAPI. **Optimized SVM (with Braden)**: conduct prevention actions for 24 patients (i.e., detection prevalence), predict 7.4 patients with HAPI (i.e., sensitivity) correctly.

A comparison of Braden Scale and the developed model can be conducted to measure the financial impacts on 100 patients. Braden Scale considers 41.96 ± 1.35% patients are at risk for HAPI development, that would equate to 41–43 patients; the addition of SVM to Braden Scale reduces the number to 24–25 patients. Therefore, the saving is between 16–19 patients who required for prevention actions per day. The NPIAP reports that the average cost of prevention is $50–$100 daily per patient in terms of time, labor, products, pressure-reducing support surfaces, and devices [46]. As a result, the developed model saves between $292,000–$346,750 per year when the daily cost is $50 per patient, whereas it saves between $584,000–$693,500 per year when the daily cost is $100 per patient, as summarized in Table 3 and graphically presented in Figure 4.

Additionally, the proposed model reduces the FPR from 41.05 ± 1.37% in the case of adopting First Braden Score only to 22.71 ± 0.37%. For instance, if there are 90 non-HAPI cases as shown in Figure 4, the Braden model predicts 36–38 as HAPI cases (i.e., type I error), while the integrated model predicts 20–21 as HAPI cases (i.e., type I error). The proposed model saves 15–18 patients’ costs, time, nurses, and resources required for preventive actions for incorrectly predicted HAPI cases.

In addition to allocation of resources and enhanced performance measures, the proposed integrated machine learning system allows for the inclusion of additional risk factors that are not considered by current assessments. The current assessments focus heavily on modifiable risk factors that can help drive interventions by reducing likelihood of prolonged pressure or supporting healthy skin/tissue. The use of machine learning can identify predictors and expand to factors that would not be feasible for the bedside nurse to find values for during day-to-day patient care, then turn them into a risk score to help drive care. Many of these risk factors are not directly modifiable in comparison to factors such as Sensory Perception or level of activity/mobility, but many of these factors can be tied into these previously identified modifiable risk factors to further drive individualization of care planning. The clinical identification that diabetes is a risk factor can translate into a heavier focus on nutritional management as well as interventions targeted at sensory perception. The use of vasopressors as a lifesaving intervention can also reduce tissue perfusion that leads to increased risk of pressure injury development; however, focusing on interventions that prevent the likelihood of prolonged pressure to the area can help mitigate the risk from the medication. Each of these factors can be tied into an existing modifiable risk factor and help further drive the interventions to individualize patient care.

Several aspects are considered in this research to achieve a generalized model that is applicable to a large number of units within the hospital system. Fifty-four different units are included in this model. However, pediatric patients, labor and delivery, and ED units are excluded. Furthermore, the optimized hyperparameters were used for 50 different experiments; in each experiment, the distribution of splitting the 80% vs. 20% was changed randomly to ensure the algorithm is robust on different distributions. Lastly, the Braden scale is used for a general adult patient population. The integrated system incorporated the Braden scale along with additional risk factors that contribute to the development of HAPI into one model.

The hybrid Braden-SVM model was deployed, automated, and integrated into EHR, which means patients’ risk factors that include the Braden Scale are collected every day and then fed to the hybrid model to classify at-risk HAPI. A daily report is sent via email at 06:30 am. The email that has the daily suspected at-risk patients is reviewed by the WOC clinical leader and WOC pressure injury preventionist. The predicted highest-risk patients have a chart review of the last 24 h performed and the current Braden Scale and plan of care developed by the Registered Nurse (RN); the WOC nurse will then contact the unit (RN) to assist with plan of care development to maximize prevention interventions that match the patient specific risk. The WOC nurse will also attend interdisciplinary rounds, when possible, to discuss pressure injury prevention plan of care with the care team and unit-based leadership. Local or systemwide barriers to prevention interventions that are identified during this round are addressed or elevated to the appropriate team. When patients with a prolonged LOS, who remain at-risk, will have continued WOC support as the plan of care changes and is adjusted; however, daily full review will move to the next highest-risk patient.

The focus of this model, from nurses’ perspective, is to target patients who will develop pressure injuries while reducing the number of false positives. The currently used validated tool, Braden Scale, provides a large number of patients that are at risk, but the percentage of patients noted to be at risk is too high to provide targeted, individualized, prevention interventions to each patient. The model that was developed reviewed a large population of HAPI cases and provided factors common to those patients while including factors noted from the Braden Scale. The percentage of patients noted to be at risk allows for targeted individualized pressure injury prevention interventions to those who would benefit most. The implementation also allows for this population to be discussed at shift safety huddles and interdisciplinary rounding. The WOC nurse team can provide guided prevention intervention discussions for this smaller population of patients.

In summary, the proposed model allows for additional risk factors to be assessed without increased burden on the care team. These factors can be quickly identified and scored using EHR. Additionally, the further narrowing of high-risk patients will allow for better allocation of resources to the highest-risk patients. Lastly, a significant improvement is clearly noticed in the performance metrics when comparing the Braden Scale alone vs. a hybrid Braden-SVM model.

This research developed an integrated Braden risk assessment and machine learning system to predict HAPI. There are some limitations to this model. Patients in the ICU/transitional setting can have prolonged LOS in the hospital. Patients identified as being at risk will likely remain at risk for the duration of their time in the healthcare system. Predicting only who will develop HAPI does not help differentiate the severity and urgency of those predicted cases as they are treated as equally likely in terms of severity. Moreover, the FPR is 22.71 ± 0.37, which would predict 22–23 HAPI cases out of 100 non-HAPI cases (i.e., type I error), which would provide preventive actions for non-HAPI cases. Furthermore, this model does not consider the weights/feature importance of each risk factor per individual suspected patient; it can provide the importance for the entire population as discussed previously. Nonetheless, the Braden risk factors might provide a direction because all of Braden’s sub-components are included in this model. Finally, the proposed model is developed based on patient records in ChristianaCare hospital, which means the model may require retraining if it is applied to a different population in another hospital.

## 7. Conclusions and Future Work

Pressure-related skin damage is a problem in the healthcare system. For the patients in care, it can lead to painful and disfiguring injuries. For the healthcare system, it can result in financial penalties, litigation, and loss of trust from the community it serves. Prevention of these injuries is the foundation of safe patient care. Several risk assessments, such as Braden Scale, are available for bedside clinicians to use to determine level of risk. However, the volume of patients identified as being at risk stretches already limited resources.

This research integrates machine learning with Braden Scale to further identify risk factors that contribute to development of pressure injuries without increased burden on the care team but cannot reasonably be assessed by the bedside clinician during patient care. The addition of machine learning enhances the sensitivity of the Braden Scale, which reduces the number of patients identified as at risk and reduces the false positive rate. This allows for better allocation of resources to patients with highest risk, and ensures automated and ongoing intervention provided to predicted patients in real time.

In the future, studies will identify not only who is at risk for developing pressure injuries but also when the pressure injuries will develop during the patient’s hospitalization. Knowing when pressure injuries are most likely to happen will allow for targeted multidisciplinary intervention when it is most needed. Furthermore, changes in patient’s position can be considered as an additional risk factor to predict HAPI in the future.

## Figures and Tables

**Table 1:** Most common risk assessment methods for predicting HAPI (adapted from [1]).

Risk Assessment Method		Braden Scale	Norton Scale	Waterlow Scale	Cubbin-Jackson Scale	Braden Q Scale	SCIPUS
Specialty		General	General/Elder Patients	General/ Orthopedics	ICU Patients	Pediatric Patients	Spinal Cord
**Advantage**	**Generalizability**	**✓**	**✓**				
	**High Sensitivity**	**✓**	**✓**	**✓**	**✓**	**✓**	**✓**
**Disadvantage**	**High False Positive Rate**	**✓**	**✓**	**✓**	**✓**	**✓**	**✓**
	**Limited Number of Risk Factors**	**✓**	**✓**	**✓**	**✓**	**✓**	**✓**
**Risk** **Factors**	**Mobility**	**✓**	**✓**	**✓**	**✓**	**✓**	**✓**
	**Activity**	**✓**	**✓**			**✓**	
	**Friction Shear**	**✓**					**✓**
	**Hygiene**				**✓**		
	**Skin Status**			**✓**	**✓**		
	**Blood Glucose Levels**						**✓**
	**Oxygenation**				**✓**	**✓**	
	**Perfusion**			**✓**		**✓**	
	**Tobacco Use**						**✓**
	**Cardiac Disease**						**✓**
	**Hemodynamics**				**✓**		
	**Respiration**				**✓**		
	**Poor Nutrition Status**	**✓**	**✓** *	**✓**	**✓**	**✓**	
	**Increase Skin moisture**	**✓**				**✓**	
	**Incontinence/** **Continence**		**✓**	**✓**	**✓**		**✓**
	**Age**			**✓**	**✓**		**✓**
	**Gender**			**✓**			
	**Sensory Perception**	**✓**				**✓**	
	**Neurological Deficit**			**✓**			
	**Abnormal Lab Blood** **Results**						**✓** **^#^**
	**Physical Condition**		**✓**				
	**Mental Condition**		**✓**				**✓**
	**Major Surgery/Trauma**			**✓**	**✓**		
	**Medications**			**✓**			
	**Past Medical** **Condition**				**✓**		
	**Respiratory Disease**						**✓**
	**Renal Disease**						**✓**
	**Weight for Height**			**✓**			

*: Modified scale, #: Albumin, and Hematocrit.

**Table 2:** Performance metrics for the proposed integrated model vs. Braden alone based on 50 different experiments.

Training Set						
		Sensitivity	Specificity	AUC	FPR	Detection Prevalence
**Proposed Model**	**Optimized SVM**	74.06 ± 0.45	77.29 ± 0.15	75.67 ± 0.20	22.71 ± 0.15	24.27 ± 0.15
**Braden Scale**	**At** **Admission**	60.62 ± 3.97	58.11 ± 0.69	59.37 ± 2.17	41.89 ± 0.69	42.82 ± 0.59
	**Before Discharge**	74.01 ± 2.48	58.98 ± 1.36	66.55 ± 1.02	41.02 ± 1.36	42.33 ±1.38
**Testing Set**						
		**Sensitivity**	**Specificity**	**AUC**	**FPR**	**Detection Prevalence**
**Proposed Model**	**Optimized SVM**	74.29 ± 1.23	77.29 ± 0.37	75.79 ± 0.58	22.71 ± 0.37	24.29 ± 0.35
**Braden Scale**	**At** **Admission**	66.90 ± 6.59	58.95 ± 1.37	62.31 ± 3.13	41.05 ± 1.37	41.96 ± 1.35
	**Before Discharge**	72.71 ± 6.89	62.54 ± 1.12	68.08 ± 3.04	37.46 ± 1.12	38.78 ± 1.12

AUC: Area Under the Curve, FPR: False Positive Rate.

**Table 3:** Financial implications for a sample of 100 patients when comparing Braden Scale alone vs. optimized SVM.

Prevention Actions Cost per Patient *	Patients Required for Prevention Actions per Day Based on the Detection Prevalence (Table 2)		Savings per Day (Number of Patients)	Savings per Year ($)
	Braden Scale Alone	Optimized SVM with Braden		
$50 per patient per day	41–43	24–25	16–19	292,000–346,750
$75 per patient per day	41–43	24–25	16–19	438,000–520,125
$100 per patient per day	41–43	24–25	16–19	584,000–693,500

* Prevention cost per HAPI patient is between $50–$100 in the United States in 2021 [46].

## Data Availability

These data were extracted from ChristianaCare Health System’s databases in Delaware, United States. It was de-identified for the purpose of this research. The data is not available for the public as it is owned by ChristianaCare.

## Supplementary Materials

The following supporting information can be downloaded at https://www.mdpi.com/article/10.3390/diagnostics13010031/s1, Table S1: Patient characteristics.
