# Supplementary material for: A Hybrid System of Braden Scale and Machine Learning to Predict Hospital-Acquired Pressure Injuries (Bedsores): A Retrospective Observational Cohort Study
Source: Diagnostics (Basel). 2022 Dec 22;13(1):31. doi: 10.3390/diagnostics13010031 (PMC9818183; doi:10.3390/diagnostics13010031)
Supplement: Supplementary file 1 [file diagnostics-13-00031-s001.zip › diagnostics-2095079-supplementary.pdf]

diagnostics

MDPI

Supplementary Table S1. Patient characteristics

			Pressure Injuries				
			Non-HAPI patients (N =15,404)		HAPI patients (N = 485)		P value
	Risk Factors	Sub-factors (if categorical)	N or mean	% or SD	N or mean	% or SD	
Demographics	Sex	Female	7233	46.96	195	40.21	**<0.01**
		Male	8171	53.04	290	59.79	
	Race	White	10045	65.21	310	63.92	0.73
		Black	4359	28.30	148	30.52	
		Multiracial	127	0.82	4	0.82	
		Other	856	5.56	23	4.74	
		Declined	17	0.11	0	0.00	
	Ethnic Group	Not Hispanic or Latino	14637	95.02	457	94.23	0.53
		Hispanic or Latino	752	4.88	28	5.77	
		Declined	15	0.10	0	0.00	
	Age	N/A	66.5	16.40	68.9	14.1	**<0.01**
Medical	American Society of Anesthesiologists (ASA) Score	Low	13086	84.95	333	68.66	**<0.01**
		Mid	742	4.82	59	12.16	
		High	1576	10.23	93	19.18	
	Emergency Department Length-of-Stay	N/A	10.15	7.63	10.09	7.86	0.36
	ICU During Encounter	Yes	4274	27.75	269	55.46	**<0.01**
		No	11130	72.25	216	44.54	
	Number of Surgeries	N/A	0.30	0.62	0.80	1.33	**<0.01**
	Palliative Orders	Yes	1888	12.26	186	38.35	**<0.01**
		No	13516	87.74	299	61.65	
	Prior Year Inpatient Visit	N/A	1.12	1.96	0.95	1.53	**0.02**
	Steroid History	Yes	5050	32.78	148	30.52	0.29
		No	10354	67.22	337	69.48	
	Transitional During Encounter	Yes	2541	16.50	170	35.05	**<0.01**
		No	12863	83.50	315	64.95	
	Number of Pressure Injuries	N/A	0.20	1.20	0.50	1.96	**<0.01**
Diagnosis	Comorbidity	N/A	7.36	4.55	7.83	4.46	**0.02**
	Depression	Yes	4988	32.38	135	27.84	**0.04**
		No	10416	67.62	350	72.16	
	Diabetes	Yes	7441	48.31	258	53.20	**0.03**
		No	7963	51.69	227	46.80	
	Renal Failure	Yes	7502	48.70	315	64.95	**<0.01**
		No	7902	51.30	170	35.05	

	**Sepsis Diagnosis**	**Yes**	1404	9.11	118	24.33	**<0.01**
		**No**	14000	90.89	367	75.67	
	**Stroke History**	**Yes**	2123	13.78	114	23.51	**<0.01**
		**No**	13281	86.22	371	76.49	
	**Pressure Injury upon Admission**	**Yes**	947	6.15	68	14.02	**<0.01**
		**No**	14457	93.85	417	85.98	
**Labs**	**Albumin (First)**	**N/A**	3.53	0.58	3.26	0.65	**<0.01**
	**Albumin (Most Recent)**	**N/A**	3.35	0.57	2.90	0.58	**<0.01**
	**Albumin (Average)**	**N/A**	3.40	0.54	2.94	0.52	**<0.01**
	**Blood Urea Nitrogen (BUN) (First)**	**N/A**	28.50	23.40	33.90	26.30	**<0.01**
	**BUN (Most Recent)**	**N/A**	26.80	22.30	30.90	25.20	**<0.01**
	**BUN (Average)**	**N/A**	27.80	19.40	33.50	19.10	**<0.01**
	**C-reactive Protein (First)**	**N/A**	25.90	66.80	44.90	86.10	**<0.01**
	**C-reactive Protein (Most Recent)**	**N/A**	13.90	47.60	24.50	58.30	**<0.01**
	**C-reactive Protein (Average)**	**N/A**	20.10	52.50	33.60	63.90	**<0.01**
	**Creatine Serum (First)**	**N/A**	1.68	2.00	2.12	2.57	**<0.01**
	**Creatine Serum (Most Recent)**	**N/A**	1.43	1.56	1.50	1.65	0.356
	**Creatine Serum (Average)**	**N/A**	1.51	1.62	1.69	1.73	**0.022**
	**Hemoglobin (First)**	**N/A**	11.91	2.61	11.72	2.59	0.107
	**Hemoglobin (Most Recent)**	**N/A**	10.90	2.79	9.88	2.02	**<0.01**
	**Hemoglobin (Average)**	**N/A**	11.09	2.21	10.13	1.85	**<0.01**
	**Lactate (First)**	**N/A**	1.31	1.87	1.82	2.30	**<0.01**
	**Lactate (Most Recent)**	**N/A**	1.14	1.63	1.55	2.07	**<0.01**
	**Lactate (Average)**	**N/A**	1.22	1.56	1.68	1.87	**<0.01**
	**Sodium (First)**	**N/A**	136.89	5.51	136.37	6.67	0.094
	**Sodium (Most Recent)**	**N/A**	138.80	4.06	138.78	4.73	0.938
	**Sodium (Average)**	**N/A**	138.58	3.98	139.01	4.48	**0.037**
	**High Mean Arterial Pressure (MAP)**	**Yes**	2877	18.68	178	36.70	**<0.01**
		**No**	12527	81.32	307	63.30	
**Medications**	**Opioids**	**Yes**	8791	57.07	384	79.18	**<0.01**
		**No**	6613	42.93	101	20.82	
	**Steroid Use**	**Yes**	8050	52.26	354	72.99	**<0.01**
		**No**	7354	47.74	131	27.01	
	**Stimuli Anesthesia**	**Yes**	2170	14.09	148	30.52	**<0.01**
		**No**	13234	85.91	337	69.48	
	**Stimuli Paralytics**	**Yes**	842	5.47	76	15.67	**<0.01**
		**No**	14562	94.53	409	84.33	
	**Stimuli Sedation**	**Yes**	2870	18.63	201	41.44	**<0.01**

		**No**	12534	81.37	284	58.56	
	**Stimuli Tracheostomy**	**Yes**	488	3.17	80	16.49	**<0.01**
		**No**	14916	96.83	405	83.51	
	**Vasopressor**	**Yes**	4306	27.95	287	59.18	**<0.01**
		**No**	11098	72.05	198	40.82	
**Medical Devices**	**Artificial Air Management**	**Yes**	2842	18.45	201	41.44	**<0.01**
		**No**	12562	81.55	284	58.56	
	**Face Mask**	**Yes**	4579	29.73	283	58.35	**<0.01**
		**No**	10825	70.27	202	41.65	
	**Nasal Cannula**	**Yes**	10398	67.50	393	81.03	**<0.01**
		**No**	5006	32.50	92	18.97	
	**Noninvasive Ventilation**	**Yes**	41	0.27	0	0.00	0.26
		**No**	15363	99.73	485	100.00	
	**Pharyngeal**	**Yes**	9	0.06	2	0.41	**<0.01**
		**No**	15395	99.94	483	99.59	
	**Room Air**	**Yes**	14374	93.31	459.000	94.64	0.25
		**No**	1030	6.69	26	5.36	
	**Ventilator**	**Yes**	2830	18.37	199.00	41.03	**<0.01**
		**No**	12574	81.63	286	58.97	
	**Feeding Tube**	**Yes**	1735	11.26	207.00	42.68	**<0.01**
		**No**	13669	88.74	278	57.32	
**Assessments**	**Blood Pressure Systolic (First)**	**N/A**	135	29.20	128	30.20	**<0.01**
	**Blood Pressure Systolic (Most Recent)**	**N/A**	125	22.20	120	23.00	**<0.01**
	**Blood Pressure Systolic (Average)**	**N/A**	127	16.40	122	14.70	**<0.01**
	**Blood Pressure Diastolic (First)**	**N/A**	78	17.60	74	18.20	**<0.01**
	**Blood Pressure Diastolic (Most Recent)**	**N/A**	72	12.50	68	13.60	**<0.01**
	**Blood Pressure Diastolic (Average)**	**N/A**	73	8.78	69	7.80	**<0.01**
	**Body Mass Index (BMI)**	**N/A**	29	25.00	26	8.46	**<0.01**
	**Count of Glasgow Coma Score (GCS) Comment of Intubated or Chemically Paralyzed**	**N/A**	899	1,126.00	2968	3,182.00	**<0.01**
	**Glasgow Coma Score (GCS) (First)**	**N/A**	14	1.73	13	2.39	**<0.01**
	**Glasgow Coma Score (GCS) (Most Recent)**	**N/A**	3	0.57	3	0.58	**<0.01**
	**Glasgow Coma Score (GCS) (Average)**	**N/A**	14	1.73	13	2.39	**<0.01**

**Patient Refusal to Change Position**	**Yes**	4466	28.99	272	56.08	**<0.01**
	**No**	10938	71.01	213	43.92	
**Pulse Oximetry (First)**	**N/A**	95.86	5.08	96	5.09	0.80
**Pulse Oximetry (Most Recent)**	**N/A**	94.96	6.58	94	90.08	0.12
**Pulse Oximetry (Average)**	**N/A**	96.02	2.13	96	1.89	**<0.01**
**Skin Abnormality on Admission**	**Yes**	15384	99.87	485	100.00	0.43
	**No**	20	0.13	0	0.00	
**Body Temperature (First)**	**N/A**	37	0.81	37	1.51	**<0.01**
**Body Temperature (Most Recent)**	**N/A**	37	0.55	37	1.42	**<0.01**
**Body Temperature (Average)**	**N/A**	37	0.26	37	0.25	**<0.01**
**Weight Loss**	**Yes**	729	0.05	34	0.07	**0.02**
	**No**	14675	0.95	451	0.93	
**Overall Braden Score (First)**	**Risky (<18)**	6393	41.50	307	63.30	**<0.01**
	**Non-Risky (≥18)**	9011	58.50	178	36.70	
**Overall Braden Score (Most Recent)**	**Risky (<18)**	5693	36.96	359	74.02	**<0.01**
	**Non-Risky (≥18)**	9711	63.04	126	25.98	
**Overall Braden Score (Average)**	**Risky (<18)**	11325	73.52	479	98.76	**<0.01**
	**Non-Risky (≥18)**	4079	26.48	6	1.24	
**Sensory Perception Status (First)**	**Completely Limited**	577	3.75	56	11.55	**<0.01**
	**Very Limited**	1031	6.69	39	8.04	
	**Slightly Limited**	3343	21.70	129	26.60	
	**No Impairment**	10453	67.86	261	53.81	
**Sensory Perception Status (Most Recent)**	**Completely Limited**	633	4.11	53	10.93	**<0.01**
	**Very Limited**	814	5.28	79	16.29	
	**Slightly Limited**	3313	21.51	136	28.04	
	**No Impairment**	1044	6.78	217	44.74	
**Sensory Perception Status (Average)**	**Completely Limited**	392	2.54	34	7.01	**<0.01**
	**Very Limited**	1488	9.66	144	29.69	
	**Slightly Limited**	8768	56.92	277	57.11	
	**No Impairment**	4756	30.88	30	6.19	
**Skin Moisture/ Circulation/ Excretion Status (First)**	**Constantly Moist**	124	0.80	7	1.44	**<0.01**
	**Often Moist**	1051	6.82	47	9.69	
	**Often Moist**	4466	28.99	201	41.44	
	**Rarely Moist**	9763	63.38	230	47.42	
**Skin Moisture/ Circulation/ Excretion Status (Most Recent)**	**Constantly Moist**	78	0.51	8	1.65	**<0.01**
	**Often Moist**	757	4.91	61	12.58	
	**Often Moist**	4051	26.30	234	48.25	

		**Rarely Moist**	10518	68.28	182	37.53	
	**Skin Moisture/ Circulation/ Excretion Status (Average)**	**Constantly Moist**	12	0.08	0.00	0.00	**<0.01**
		**Often Moist**	1893	12.29	139	28.66	
		**Often Moist**	8097	52.56	323	66.60	
		**Rarely Moist**	5402	35.07	23	4.74	
	**Activity Status (First)**	**Bedfast**	3429	22.26	172	35.46	**<0.01**
		**Chairfast**	1877	12.19	87	17.94	
		**Walks Occasionally**	6840	44.40	174	35.88	
		**Walks Frequently**	3258	21.15	52	10.72	
	**Activity Status (Most Recent)**	**Bedfast**	2362	15.33	182	37.53	**<0.01**
		**Chairfast**	2418	15.70	146	30.10	
		**Walks Occasionally**	7619	49.46	137	28.25	
		**Walks Frequently**	3005	19.51	20	4.12	
	**Activity Status (Average)**	**Bedfast**	3690	23.95	276	56.91	**<0.01**
		**Chairfast**	5384	34.95	179	36.91	
		**Walks Occasionally**	6145	39.89	30	6.19	
		**Walks Frequently**	185	1.20	0.00	0.00	
	**Mobility Status (First)**	**Completely Immobile**	692	4.49	49	10.10	**<0.01**
		**Very Limited**	2392	15.53	125	25.77	
		**Slightly Limited**	6142	39.87	195	40.21	
		**No Limitations**	6178	40.11	116	23.92	
	**Mobility Status (Most Recent)**	**Completely Immobile**	819	5.32	66	13.61	**<0.01**
		**Very Limited**	1857	12.06	131	27.01	
		**Slightly Limited**	6353	41.24	220	45.36	
		**No Limitations**	6375	41.39	68	14.02	
	**Mobility Status (Average)**	**Completely Immobile**	770	5.00	72	14.85	**<0.01**
		**Very Limited**	4199	27.26	290	59.79	
		**Slightly Limited**	8744	56.76	121	24.95	
		**No Limitations**	1691	10.98	2	0.41	
	**Nutrition Status (First)**	**Very Poor**	596	3.87	36	7.42	**<0.01**
		**Probably Inadequate**	2293	14.89	112	23.09	
		**Adequate**	9477	61.52	269	55.46	
		**Excellent**	3038	19.72	68	14.02	
	**Nutrition Status (Most Recent)**	**Very Poor**	579	3.76	29	5.98	**<0.01**
		**Probably Inadequate**	1345	8.73	84	17.32	
		**Adequate**	11084	71.96	326	67.22	
		**Excellent**	2396	15.55	46	9.48	

	**Nutrition Status (Average)**	**Very Poor**	350	2.27	17	3.51	**<0.01**
		**Probably Inadequate**	6084	39.50	349	71.96	
		**Adequate**	8927	57.95	119	24.54	
		**Excellent**	43	0.28	0.00	0.00	
	**Friction and Shear Status (First)**	**Problem**	821	5.33	47	9.69	**<0.01**
		**Potential Problem**	4745	30.80	210	43.30	
		**No Apparent Problem**	9838	63.87	228	47.01	
	**Friction and Shear Status (Most Recent)**	**Problem**	1085	7.04	163	33.61	**<0.01**
		**Potential Problem**	4767	30.95	203	41.86	
		**No Apparent Problem**	9552	62.01	119	24.54	
	**Friction and Shear Status (Average)**	**Problem**	2100	13.63	224	46.19	**<0.01**
		**Potential Problem**	8973	58.25	257	52.99	
		**No Apparent Problem**	4331	28.12	4	0.82	

SD: Standard deviation, Gray color: Risk Factor selected by machine learning, Bold P value: statistically significant (< 0.05), N/A: Continuous factors; no sub-categories (t-test, otherwise it is categorical: Chi-square test), First: risk factors/diagnosis upon admission, Last: Most recent diagnosis before discharge, Average: the average of all observed values during visit.
